# Together and apart: inhibition of DNA synthesis by connexin-43 and its relationship to transforming growth factor β

**DOI:** 10.3389/fphar.2013.00090

**Published:** 2013-07-19

**Authors:** Maya M. Jeyaraman, Robert R. Fandrich, Elissavet Kardami

**Affiliations:** ^1^Institute of Cardiovascular Sciences, St. Boniface Research Centre, University of ManitobaWinnipeg, MB, Canada; ^2^Department of Physiology, University of ManitobaWinnipeg, MB, Canada; ^3^Department of Human Anatomy and Cell Sciences, University of ManitobaWinnipeg, MB, Canada

**Keywords:** connexin-43, phosphorylation, cell proliferation, transforming growth factor β, cardiomyocytes

## Abstract

The membrane and channel protein connexin-43 (Cx43), as well as the cytokine transforming growth factor (TGF) β, suppress proliferative growth in cardiomyocytes and other cell types. Previously we showed that the inhibitory effect of Cx43 is canceled when Cx43 becomes phosphorylated at serine (S) 262 in response to mitogen stimulation. We have now asked if the TGFβ-triggered inhibition of DNA synthesis is associated with changes in Cx43 phosphorylation at S262. Conversely, we investigated if inhibition of DNA synthesis by overexpressed Cx43 is dependent on engaging TGFβ signal transduction. We report that TGFβ acutely prevented mitogen-induced Cx43 phosphorylation at S262, while chronic inhibition of TGFβ signal transduction raised baseline levels of endogenous phospho-S262-Cx43 without affecting total Cx43. Inhibition of baseline TGFβ signal transduction through (a) inhibiting TGFβ receptor I (TGFβRI) with SB431542, (b) inhibiting TGFβ receptor II (TGFβRII) by overexpressing dominant-negative (DN) TGFβRII, (c) inhibiting the downstream signaling mediator Smad2 by overexpressing DN Smad2, each separately increased baseline cardiomyocyte DNA synthesis, but could not reverse DNA synthesis inhibition by overexpressed Cx43. It is suggested that inhibition of cardiomyocyte DNA synthesis by TGFβ/TGFβRI/II/phospho-Smad2 signaling is mediated, at least in part, by reducing endogenous phospho-S262-Cx43 levels.

## INTRODUCTION

Cardiomyocytes, the contractile functional units of the heart pump, are proliferative during the embryonic and early neonatal stages. Subsequently cardiac increases in mass and size occur mainly by increased size of individual myocytes (hypertrophy) rather than cell proliferation (Ahuja et al., 2007). Ischemic heart disease and myocardial infarction cause damage and loss of functional myocardium, which is replaced mainly by scar tissue, resulting in maladaptive remodeling and heart failure. Endogenous capacity for regeneration after extensive injury resulting in cell death is inadequate to replace lost cardiac tissue; nevertheless, a small percentage of adult cardiomyocytes maintain the capacity to enter the cell cycle (Bergmann et al., 2009), and this percentage increases after myocardial infarction (Senyo et al., 2013), indicative of an attempt for a regenerative response. To improve cardiac regeneration after injury it is important to identify factors and mechanisms stimulating or inhibiting cardiomyocyte proliferation. This understanding can provide strategies for stimulating or dis-inhibiting cardiomyocyte proliferation as may be needed during cardiac repair after myocardial infarction.

Overexpression as well as knock-down studies have shown that the membrane and gap junction channel phosphoprotein connexin-43 (Cx43) inhibits DNA synthesis in cardiomyocytes and several other cell types (Doble et al., 2004; Kardami et al., 2007; Zhang et al., 2008; Matsuyama and Kawahara, 2009). The mechanism by which Cx43 affects cell proliferation includes significant effects on gene expression; and does not require the channel-forming ability of the molecule (Kardami et al., 2007). It is important to note that the ability of Cx43 to inhibit cell proliferation is regulatable: mitogen-induced phosphorylation of Cx43, or the C-terminal of Cx43 (Cx43-CT) at S262, was shown to cancel their inhibitory effect on DNA synthesis (Doble et al., 2004; Dang et al., 2006). Mitogens such as fibroblast growth factor 2 (FGF-2) stimulate cardiomyocyte proliferation, as well as Cx43 phosphorylation at S262.

The proliferative action of FGF-2 is counteracted by another multifunctional protein, transforming growth factor (TGF) β, which inhibits cardiomyocyte proliferation (Kardami, 1990; Sheikh et al., 2004). Proliferative growth suppression by Cx43 may be linked to TGFβ signaling: Cx43 was reported to potentiate TGFβ signaling in the atria-derived cardiomyocyte cell line HL-1, by competing with the downstream mediator of TGFβ signal transduction, Smad2, for binding to tubulin (Dai et al., 2007). This competitive binding released Smad2 from microtubules, thus making it available for phosphorylation by TGFβ receptor I (TGFβRI), followed by nuclear translocation, and activation of TGFβ responsive genes (Dai et al., 2007). In addition, TGFβ is known to upregulate Cx43 expression in a number of cell types including epithelial cells and vascular smooth muscle cells, and this upregulation may contribute to inhibition of DNA synthesis (Rama et al., 2006; Tacheau et al., 2008).

In the present study, we addressed the potential relationship between Cx43 and TGFβ-mediated inhibition of cardiomyocyte DNA synthesis. Our data showed that TGFβ signaling inhibited the growth factor-induced phosphorylation of endogenous Cx43 at S262. On the other hand, inhibition of cardiomyocyte DNA synthesis by overexpressed Cx43 did not require downstream activation of TGFβ-related signals such as TGFβRI, TGFβRII, or Smad2. Overall our data suggest that Cx43-mediated inhibition is downstream of early TGFβ signal transduction, and that the mechanism of TGFβ-triggered inhibition of cardiomyocyte DNA synthesis includes downregulation of endogenous pS262-Cx43.

## MATERIALS AND METHODS

### ANIMALS

One-day-old Sprague Dawley rat pups were obtained from the Central Animal Care Facility at the University of Manitoba. This study was carried out in strict accordance with the recommendations in the Guide for the Care and Use of Laboratory Animals by the US National Institutes of Health (NIH Publication No. 85-23, revised 1996). Approval for use of rat pups was obtained by the Protocol Management and Review Committee of the University of Manitoba.

### REAGENTS

Rabbit polyclonal antibodies recognizing: (i) total Cx43 (phosphorylated as well as unphosphorylated), were raised in-house against a synthetic peptide containing Cx43 residues 367–382; the immune serum was used at 1:10,000 dilution for western blotting, (ii) anti-phospho-(p) 262-Cx43 antibodies were purchased from Santa Cruz Biotechnology (CA, USA), as a 200-μg/ml solution, and were used at 1:1000 dilution. These antibodies have been characterized and described previously (Doble et al., 2004; Srisakuldee et al., 2009). Anti-bromodeoxyuridine (BrdU) antibodies (GE Biosciences) were used at 1:1000 dilution. Anti-phospho-(p)Smad2(Ser465/467), or anti-Smad2 antibodies were purchased from Upstate or Cell Signaling Technology (MA, USA), respectively and used at 1:1000 dilution. Mouse monoclonal anti-α-actinin (1:200) and rabbit anti-actin (1:1000) antibody were from Sigma (USA). Goat anti-mouse and anti-rabbit HRP (horse radish peroxidase) secondary antibodies, were obtained from Bio-Rad (CA, USA) and used as per manufacturer’s instructions. The TGFβRI inhibitor SB431542 was purchased from Tocris Bioscience (Bristol, UK). TGFβ1 was purchased from R&D Systems (USA), and used at 5 ng/ml. Recombinant 18 kDa FGF-2 was produced in-house as we have described (Jiang et al., 2002, 2004), and used at 10 ng/ml. Adenoviral vectors expressing wild type-Cx43, mutant S262A-Cx43 or truncated Cx43-CT (residues 247–382), have been described previously (Doble et al., 2004; Kardami et al., 2007), and were used at low titers (2 m.o.i; multiplicity of infection), achieving modest overexpression, namely a two- to threefold increase in total Cx43 (Srisakuldee et al., 2009). The adenoviral vector expressing TGFβRII-dominant-negative (DN) has been described (Sheikh et al., 2004) and was used at 50 m.o.i. Adenoviral vectors expressing DN Smad2-DN, or Smad3-DN have been described in (Uemura et al., 2005) and were generous gifts from Dr. Rebecca Wells (University of Pennsylvania School of Medicine, PA, USA); they were used at 100 m.o.i.

### WESTERN BLOT ANALYSIS

Lysates were analyzed on 10% polyacrylamide gels, at 10 μg protein/lane, as described previously (Srisakuldee et al., 2009). Broad range (6.5–200 kDa) molecular mass standards (Bio-Rad) were used in all analyses. Protein concentration was determined by the bicinchoninic acid (BCA) protein assay reagent (Pierce) followed by spectrophotometry. The proteins on the gel were electrophoretically transferred to polyvinylidene difluoride membranes. Antigen–antibody complexes were visualized using enhanced chemiluminescence (ECLplus), from Amersham Pharmacia. Densities of western blot bands were determined using the Bio-Rad Model GS-800 densitometer with Molecular Analyst software (Bio-Rad). Band densities were adjusted based on the density of corresponding loading controls.

### NEONATAL RAT VENTRICULAR MYOCYTE CULTURES

Cardiomyocytes were isolated from the ventricles of 1-day-old rat pups according to standard procedures (Doble et al., 1996). For studies on DNA synthesis, myocytes were plated on collagen-coated coverslips at 400,000 cells/35 mm well, in the presence of 10% bovine calf serum supplemented with FGF-2 (10 ng/ml) and routinely maintained in this type of medium in experiments using adenoviral vector gene transfer. In some experiments, myocytes were placed in a low serum medium (0.5% bovine calf serum supplemented with 0.5% bovine serum albumin (BSA), 1% penicillin/streptomycin, 0.04% vitamin C, 0.1% insulin, 0.1% transferrin/selenium) for 48 h, followed by stimulation with FGF-2 (10 ng/ml, 30 min) in the presence or absence of 15 min pre-treatment with TGFβ (5 ng/ml).

### BROMODEOXYURIDINE LABELING INDEX

As described previously (Doble et al., 2004). Briefly,cardiomyocytes cultured on coverslips were incubated with BrdU (3 μg/ml) for 8–12 h prior to the termination of various experiments. Coverslips were then fixed with 1% paraformaldehyde for 15 min in the cold, and then treated with 0.07 M NaOH for 2 min at room temperature. Labeling for sarcomeric α-actinin (exclusively cytosolic as well as cardiomyocyte-specific), for BrdU (to identify nuclei synthesizing DNA), and Cx43 (to ascertain Cx43 overexpression after transfection) was achieved using mouse monoclonal antibodies against α-actinin, against BrdU, and rabbit polyclonal antibodies against Cx43. Coverslips were also counterstained for DNA, with Hoechst 33342. A minimum of 24 randomly selected visual fields, distributed in three coverslips, were observed under epifluorescence optics, photographed, and individually scored for numbers of BrdU-positive cardiomyocyte nuclei, over total cardiomyocyte nuclei (BrdU labeling index).

### STATISTICS

The GraphpadStat and SigmaStat software programs were used for data analysis. Data are presented as mean ± SEM (standard error of the mean). Statistical analysis was performed using either one-way ANOVA to compare more than two groups or two-way ANOVA to compare more than two groups with two independent variables. *P* < 0.05 was considered statistically significant. *P* < 0.01 was considered statistically very significant.

## RESULTS

### EFFECT OF TGFβ ON RELATIVE CARDIOMYOCYTE pS262-Cx43 LEVELS

Our previous studies have demonstrated that cardiomyocytes maintained in culture under growth-stimulating conditions, are nevertheless subjected to a degree of mitotic suppression by “baseline TGFβ,” representing TGFβ present in serum, and/or made by myocytes and the small amount of contaminating fibroblasts, in culture. Inhibiting this baseline TGFβ signaling potentiated the ability of growth factors such as FGF-2 to stimulate cardiomyocyte proliferation (Doble et al., 2004; Sheikh et al., 2004). As we have found a positive relationship between growth factor-induced Cx43 phosphorylation at S262, and the ability of growth factors to stimulate cardiomyocyte proliferation, we asked whether inhibition of baseline TGFβ signal transduction would influence levels of endogenous pS262-Cx43, in culture.

**FIGURE 1:**
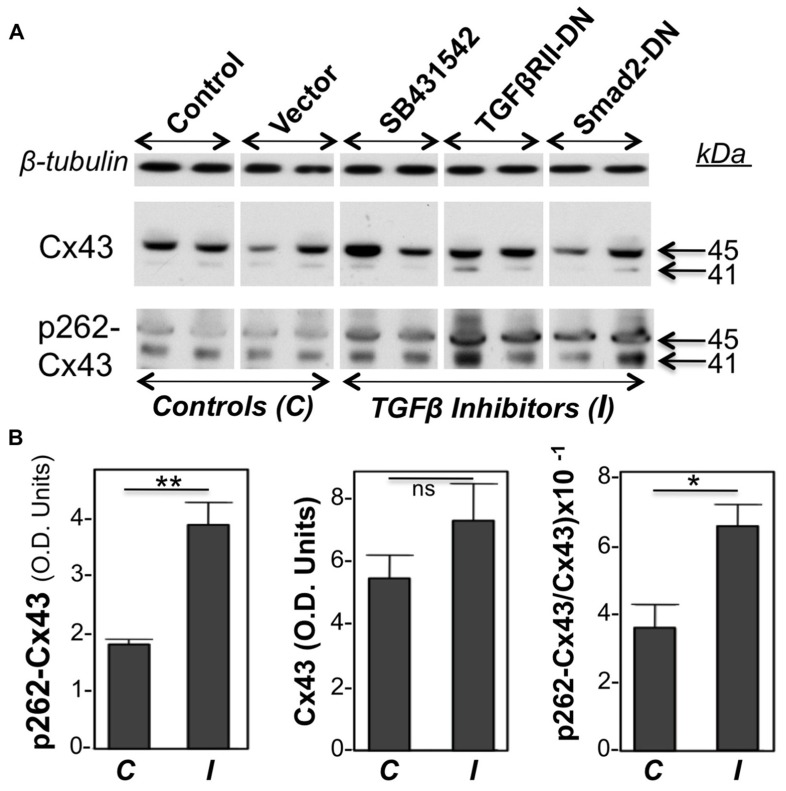
**FIGURE 1. Inhibition of TGFβ signaling increases baseline cardiomyocyte Cx43 phosphorylation at S262.(A)** Western blots of cardiomyocyte lysates probed for total Cx43 (Cx43), pS262-Cx43, and β-tubulin (loading control), as indicated. Samples shown represent five groups, including two types of controls *(C)*, such as myocytes subjected to no treatment (Control), or exposed to an empty adenoviral vector (Vector); the two controls produced similar data. The remaining three groups were treated with TGFβ signaling inhibitors *(I)*, such as SB431542 (20 μM) and adenoviral vectors expressing TGFβRII-DN or Smad2-DN, as indicated. All three groups showed increased signal for pS262-Cx43, compared to control groups. **(B)** Combined densitometry data (optical density, OD units) comparing relative pS262-Cx43, total Cx43, or the ratio of pS262-Cx43/total Cx43 between control, *C* (*n* = 4), and *I*-treated groups (*n* = 6). **, *, and ns denote *P* < 0.01, *P* < 0.05, and *P* < 0.05.

Primary cultures of neonatal cardiac myocytes, placed under conditions stimulating proliferative growth (10% bovine calf serum, plus 10 ng/ml FGF-2) were exposed to a pharmacological inhibitor of TGFβRI (SB431542), or subjected to overexpression of DN versions of either TGFβRII or Smad2, through adenovirally mediated transient gene transfer. One day following these manipulations cell lysates were analyzed for total as well as pS262-Cx43 by western blotting. Two types of control cultures were used: in the first, cells were kept in growth medium without any treatment, while in the second cells were treated with an empty adenoviral vector. As shown in **Figure 1**, both types of controls elicited signals of similar intensity for pS262-Cx43, as well as total Cx43, indicating that the adenoviral vector had no effect on Cx43. Treatment of cardiomyocytes with inhibitors of TGFβ signal transduction elicited a significant increase in pS262-Cx43 (**Figure 1**), but had no significant effect on total Cx43.

Probing with antibodies recognizing total Cx43 showed that the majority of Cx43 migrated at 45 kDa, representing extensively phosphorylated Cx43, with a faint signal near 41 kDa, representing unphosphorylated or minimally phosphorylated Cx43. This pattern is typical for cardiomyocytes (Doble et al., 2004; Srisakuldee et al., 2009). Probing with anti-pS262-Cx43 detected bands migrating near 41 as well as 45 kDa, indicating that, under “growth stimulation” conditions, phosphorylation at the S262 site can occur, respectively, on Cx43 that lacks substantial phosphorylation at other sites, as well as on Cx43 extensively phosphorylated at other sites. Please note that any shift in motility that might be caused by phosphorylation at a single site (S262) would not be detectable by standard one-dimensional electrophoresis as used here. Inhibition of TGFβ signal transduction upregulated both the faster and slower migrating pS262-Cx43 bands. **Figure 1B** is showing densitometric results representing the sum of Cx43 or pS262-Cx43 bands.

**FIGURE 2:**
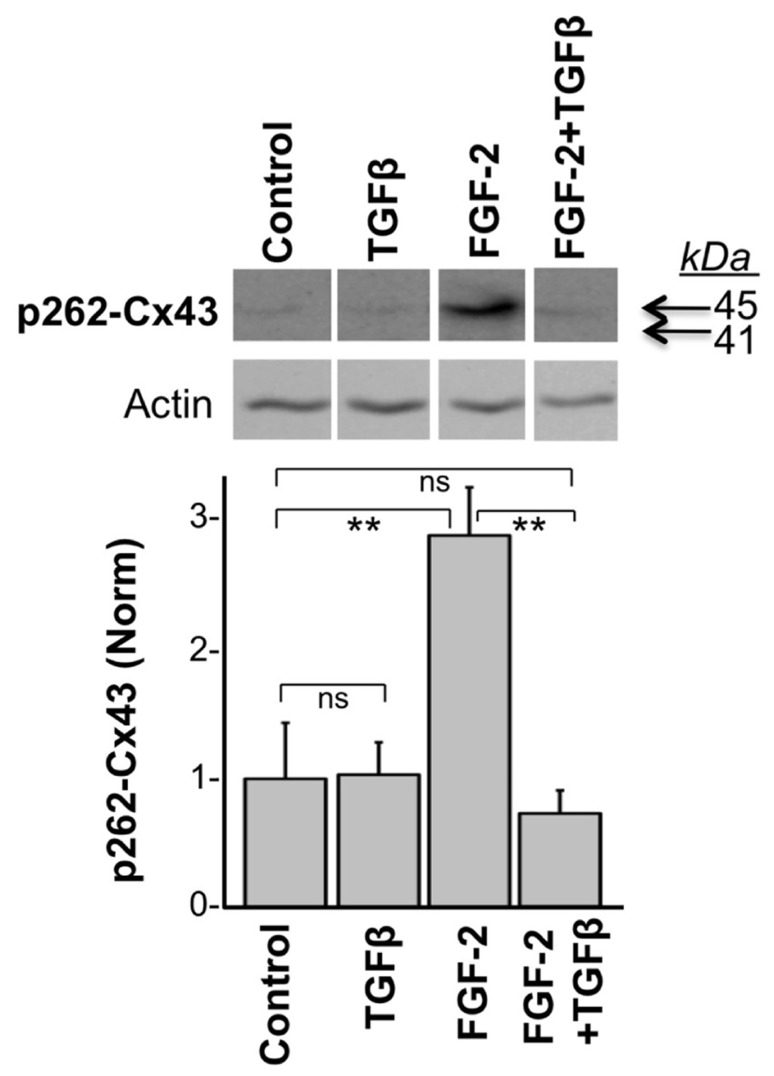
**FIGURE 2. TGFβ prevents the FGF-2-induced stimulation of Cx43 phosphorylation at S262.** Representative western blots of cardiomyocyte cultures stimulated with FGF-2 (10 ng/ml) in the absence or presence of TGFβ pre-treatment for 15 min, as indicated. Corresponding normalized densitometry-based data are also included. ** and ns correspond to *P* < 0.01, or *P* > 0.05; *n* = 3.

In another experiment, we tested the effect of TGFβ on the acute FGF-2-induced stimulation of Cx43 phosphorylation at S262. To minimize baseline protein kinase C (PKC) activity, and thus baseline levels of pS262-Cx43, cardiomyocytes were kept in low serum (0.5% fetal bovine serum) for 48 h before stimulation, as described previously (Doble et al., 2004). Myocytes were then subjected or not to a brief 15-min pre-incubation with TGFβ (5 ng/ml), and then stimulated with FGF-2 (10 ng/ml) for 30 min. FGF-2 significantly increased the anti-pS262-Cx43 signal, migrating at 45 kDa, in the absence of TGFβ pre-treatment, as expected from previous studies (Doble et al., 2004); **Figure 2**. The stimulatory effect of FGF-2 was, however, completely prevented in cells pre-treated with TGFβ; **Figure 2**. The faster migrating pS262-Cx43 observed in the previous experiment (**Figure 1**) was not detectable in cultures kept in low serum and may be a characteristic of cultures grown under conditions promoting proliferative growth.

### EFFECT OF INHIBITION OF TGFβ SIGNAL TRANSDUCTION ON GROWTH SUPPRESSION BY OVEREXPRESSED Cx43

To examine the hypothesis that growth suppression by Cx43 is mediated by downstream activation of TGFβ signal transduction, cardiomyocyte cultures were subjected to Cx43 or Cx43-CT overexpression, in a background of pharmacological (SB431542) TGFβRI inhibition. BrdU labeling index (proportion of cardiomyocyte nuclei incorporating BrdU, over total number of cardiomyocyte nuclei) was determined 2 days later, as a measure of relative DNA synthesis ability. We have established in previous studies that cardiomyocyte labeling index determinations are mirrored by, and are therefore representative of, cell number determinations (Kardami, 1990; Pasumarthi et al., 1996). The BrdU labeling index of control groups, maintained in growth-stimulating conditions (FGF-2-supplemented serum), varied between 0.25 and 0.30 in different primary cardiomyocyte preparations.

**FIGURE 3:**
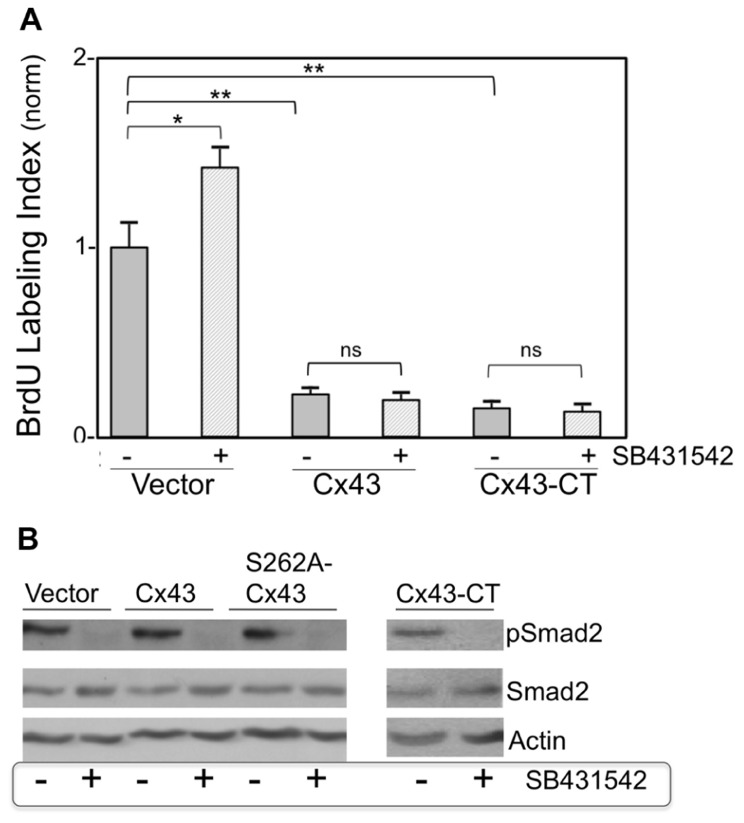
**FIGURE 3. (A)** Pharmacological inhibition of TGFβRI does not affect suppression of DNA synthesis by overexpressed Cx43 or Cx43-CT. The *y*-axis shows normalized BrDU labeling index of cardiomyocytes transduced either with an empty adenoviral vector, or adenoviral vectors expressing Cx43, or channel-domain-deficient Cx43-CT, and subjected (or not) to SB431542 treatment (indicated by + or –, respectively). Comparisons between groups are indicated by brackets (*P < 0.05, two-way ANOVA, *n* = 24; data are mean + SEM). **(B)** Smad2 activity (pSmad2), or its inhibition by SB431542, are not affected by overexpression of Cx43, S262A-Cx43, or Cx43-CT. Western blots (representative of *n* = 2) showing the effect of ±SB431542, on pSmad2, and total Smad2, in myocytes transduced either with an empty adenoviral vector (Vector) or with adenoviral vectors expressing Cx43, S262-Cx43, or Cx43-CT, as indicated. Expression of the transduced genes was verified by western blotting or immunofluorescence (data not shown).

**Figure 3A** compares normalized BrdU labeling index between the various groups, where the value for the control group, subjected to treatment with an empty adenoviral vector (Vector), was arbitrarily defined as 1. In the absence of Cx43 or Cx43-CT overexpression, inhibition of TGFβRI by SB431542 elicited a significant increase in BrdU incorporation (*P* < 0.05), as anticipated from successful inhibition of baseline TGFβ signal transduction (Sheikh et al., 2004). In the absence of SB431542, overexpression of Cx43, or Cx43-CT elicited a robust inhibition of cardiomyocyte BrdU labeling index, compared to Vector-treated controls, in agreement with our previous report that inhibition of cardiomyocyte DNA synthesis by Cx43 does not require the channel-forming portion of the molecule (Kardami et al., 2007). The presence of SB431542 was unable to reverse/prevent the inhibitory effects of Cx43, or Cx43-CT overexpression, indicating that suppression of DNA synthesis by Cx43, or Cx43-CT overexpression does not depend on activation of TGFβRI. In fact it would appear that Cx43, or Cx43-CT, overexpression, blocked the ability of SB431542 to increase baseline cardiomyocyte DNA synthesis. This suggested the possibility that Cx43 overexpression may have somehow blunted the ability of SB431542 to inhibit TGFβRI. If that were the case, SB431542 would not be able to prevent the downstream phosphorylation and activation of Smad2 (pSmad2). We therefore tested the status of Smad2 activation (pSmad2, phosphorylated at serines 465/467) in response to SB431542, Cx43-, Cx43-CT, and a Cx43 phosphorylation mutant (S262A-Cx43). Representative data are shown in **Figure 3B**. SB431542 eliminated baseline Smad2/3 activation under control conditions, validating its ability to block downstream TGFβ-TGFR1 signal transduction. The ability of SB431542 to prevent baseline Smad2/3 activation was maintained in the presence of Cx43, Cx43-CT, and S262A-Cx43 overexpression. Furthermore, none of the overexpressed proteins (Cx43, Cx43-CT, S262A-Cx43) appeared to affect baseline levels of pSmad2/3; no discernible changes were observed in total Smad2/3 in any of the groups tested.

**FIGURE 4:**
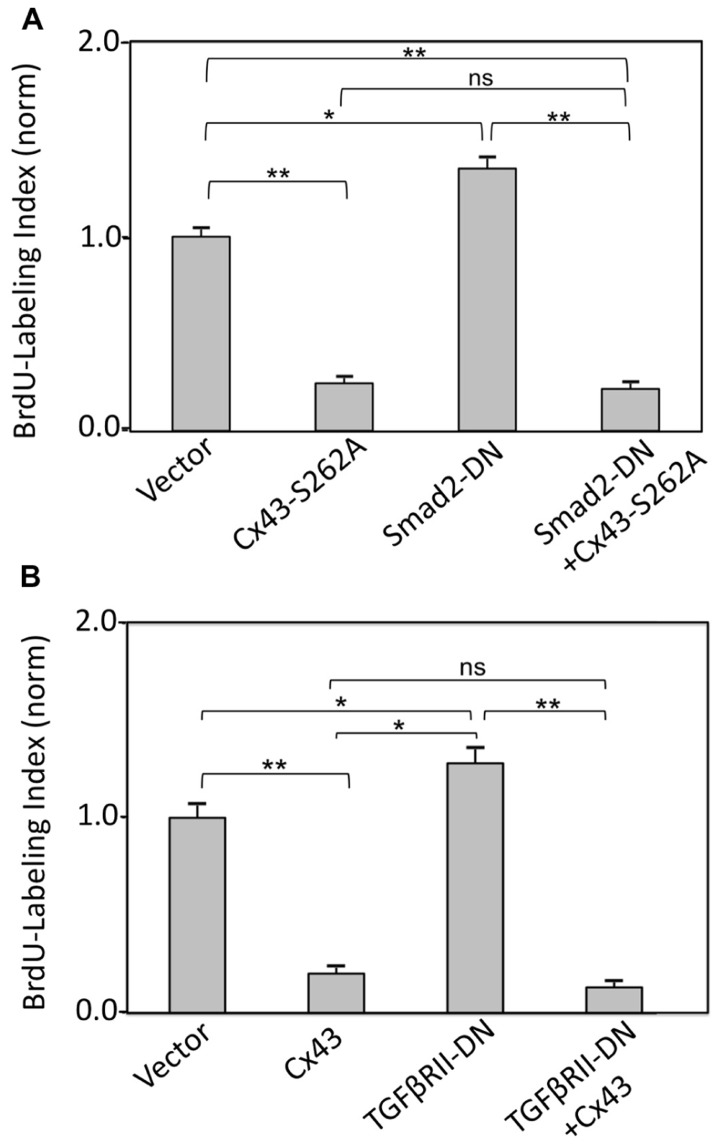
**FIGURE 4. (A)** Inhibition of TGFβRII does not affect suppression of DNA synthesis by Cx43 overexpression. The *y*-axis shows normalized BrdU labeling index of cardiomyocytes transduced with an empty adenoviral vector (Vector) or adenoviral vectors expressing Cx43, TGFβRII-DN, alone or in combination, as indicated. **(B)** Inhibition of Smad2 does not affect suppression of DNA synthesis by overexpressed S262A-Cx43. The *y*-axis shows normalized BrdU labeling index of cardiomyocytes transduced with an empty adenoviral vector (vector) or adenoviral vectors expressing S262A-Cx43, or Smad2-DN, or a combination of both. Data are shown as mean + SEM; brackets indicate comparisons between groups. **, *, and ns correspond to *P* < 0.01, *P* < 0.05, *P* > 0.05. Expression of the transduced genes was verified by western blotting (data not shown).

The effect of directly inhibiting Smad2 (by expressing a cDNA for Smad2-DN) on cardiomyocyte BrdU labeling index, in the absence or presence of Cx43 or S262A-Cx43 overexpression, was also examined. Expression of Smad2-DN significantly increased cardiomyocyte labeling index over control cells (**Figure 4A**). As expected, expression of S262A-Cx43 decreased BrdU incorporation significantly compared to controls; expression of wild type Cx43 exerts a similar inhibitory effect (**Figure 3A**). The effect of S262A-Cx43, as well as wild type Cx43 remained unchanged in the presence of Smad2-DN (**Figure 4A** and unpublished observations). The inability of Smad2-DN to reverse the inhibitory effect of S262A-Cx43 may be linked to the inability of the mutant Cx43 to become phosphorylated at amino-acid 262, providing an irreversible signal.

Inhibition of TGFβRII by overexpressing TGFβRII-DN significantly increased BrdU labeling index in cardiomyocytes, as we reported previously (Sheikh et al., 2004). This effect was prevented in the presence of Cx43 overexpression (**Figure 4B**).

Taken together, our findings with inhibitors of early TGFβ signal transduction are consistent with a scenario where Cx43-mediated growth suppression occurs downstream of TGFβ/TGFβRII/TGFβRI/pSmad2 signaling.

**FIGURE 5:**
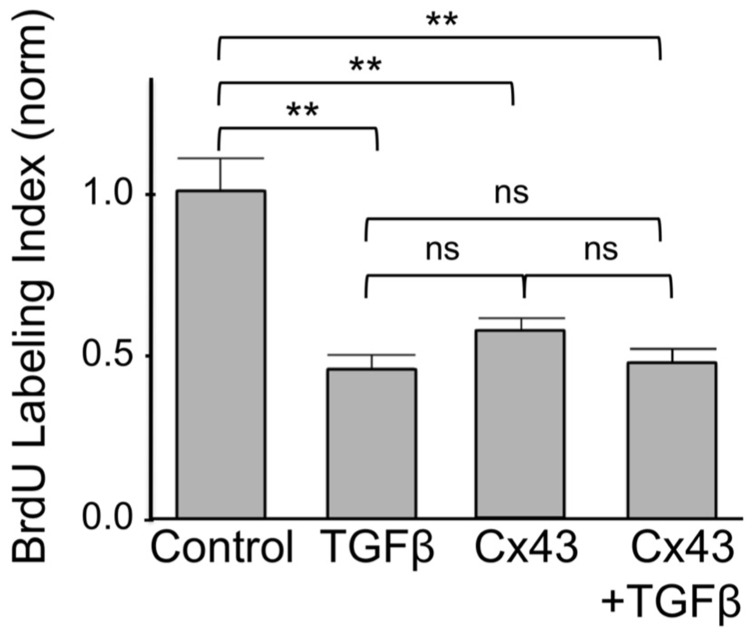
**FIGURE 5. Suppression of DNA synthesis by Cx43 overexpression is not additive to that by TGFβ.** The *y*-axis shows normalized BrdU labeling index of untreated cardiomyocytes (Control); cardiomyocytes treated with TGFβ; subjected to Cx43 overexpression; or treated with TGFβ in the presence of Cx43 overexpression, as indicated. Absolute BrdU labeling index was at 0.3. Data are shown as mean ± SEM. Brackets show comparisons between groups. ** and ns correspond to *P* < 0.01, or *P* > 0.05.

Finally we asked if inhibition of cardiomyocyte DNA synthesis by Cx43 was additive to that by TGFβ. As shown in **Figure 5**, addition of TGFβ or overexpression of Cx43, both exerted a significant inhibitory effect on BrdU labeling index, assessed 1 day after treatment. The degree of inhibition by TGFβ was not significantly different to that by Cx43. The extent of inhibition of BrdU incorporation by combined use of TGFβ and Cx43 overexpression was not significantly different to that of either inhibitor alone. The absence of an additive effect on DNA synthesis inhibition suggests that TGFβ-triggered signals, and Cx43-mediated signals, are components of the same pathway, where, as indicated by our results shown in the previous **Figures (1–5**), Cx43-triggered signals are downstream of early TGFβ signal transduction.

**FIGURE 6:**
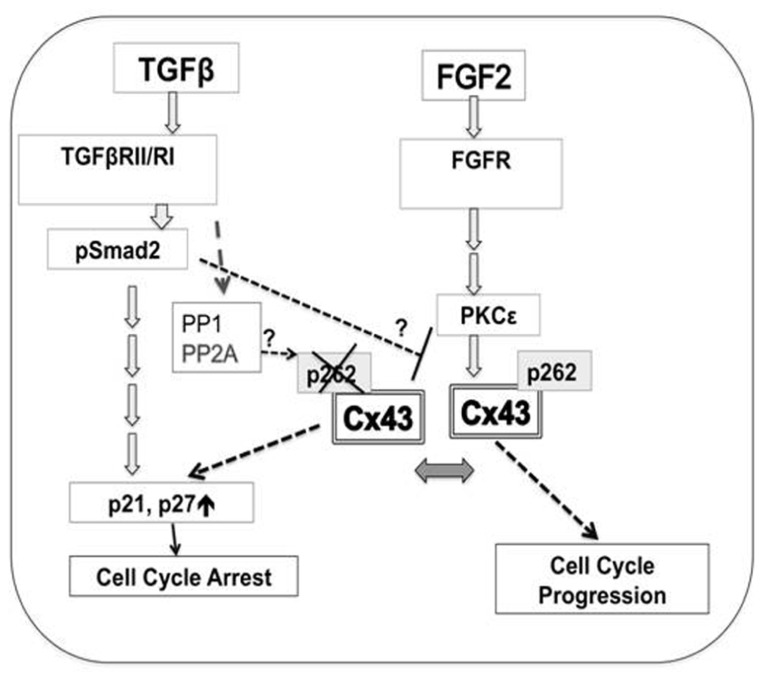
**FIGURE 6. A hypothetical model for the regulation of cardiomyocyte proliferation by Cx43 phosphorylation at S262.** Mitogen (FGF-2)-triggered signaling includes activation of cognate receptor (FGFR, fibroblast growth factor receptors), downstream activation of PKCε, followed by increased interaction between PKCε and Cx43, and phosphorylation of Cx43 at S262. This phosphorylation event blocks the ability of Cx43 to act as a suppressor of DNA synthesis, and allows FGF-2 signaling to promote cell cycle progression. Early TGFβ-triggered signaling including TGFβRII/RI and Smad2/3 activation results in decreased levels of pS262-Cx43, allowing Cx43 to act as a growth suppressor. TGFβ signaling may: activate phosphatases such as PP1, or PP2A, to affect Cx43 dephosphorylation; prevent the FGF-2-induced PKCε activation, and/or prevent PKCε/Cx43 interaction and thus Cx43 phosphorylation. These possibilities are indicated by question marks. Cx43-, as well as TGFβ-mediated inhibition of cell proliferation is achieved via downstream activation of cell cycle inhibitors p21/p27.

Experimental evidence presented here in combination with previous work pointed to a hypothetical scenario where phosphorylation of Cx43 at S262 allows this protein to act as a switch between pro-mitotic and anti-mitotic signaling, as illustrated in **Figure 6**.

## DISCUSSION

Connexin-43 is constitutively phosphorylated at multiple sites, ensuring correct trafficking of this protein to the plasma membrane as well as assembly of connexons and channel functionality (Solan and Lampe, 2009). In addition to the constitutively phosphorylated sites, specific sites at the C-terminal tail of Cx43 can become phosphorylated in response to growth factor- or oncogene-linked signaling, preventing Cx43 from suppressing cell proliferation. For example, the src-targeted tyrosines 247 and 265 can affect the ability of Cx43 to inhibit proliferation of glioma cells (Herrero-Gonzalez et al., 2010); serines 255, 262, 279, 282 are reported as targets of mitogen-activated protein kinase pathways, and, their phosphorylation allows for platelet-derived growth factor-triggered mitotic stimulation of vascular smooth muscle cells (Johnstone et al., 2012). In cardiomyocytes, the FGF-2-induced mitotic stimulation is accompanied by increased phosphorylation of Cx43 at S262, mediated by PKCε (Doble et al., 2000, 2004). To understand the role of phosphorylation at S262 on the ability of Cx43 to suppress growth, in previous studies we used Cx43 phosphorylation mutants to simulate either constitutive phosphorylation (S262-to-aspartate (D) substitution) or lack of phosphorylation (S262A) at the S262 site. The ability of Cx43 to suppress DNA synthesis was found to be maximal in cells expressing S262A-Cx43 but absent in cells expressing S262D-Cx43 (Doble et al., 2004; Dang et al., 2006). Overall our previous work indicated that pS262-Cx43 is “permissive” for DNA synthesis, allowing cells to progress through the cell cycle in response to growth factor stimulation. One may therefore consider the notion that conditions and factors known to inhibit cell proliferation may actively prevent the phosphorylation of Cx43 at specific sites. TGFβ is one such factor, known for its cytostatic properties in many situations (Massague, 2000). Because TGFβ signaling counteracts the stimulatory effect of FGF-2 on cardiomyocytes DNA synthesis (Kardami, 1990), we hypothesized that TGFβ may promote the “growth inhibitory” state of Cx43, by preventing its phosphorylation at S262. This question was addressed in both an acute as well as a more “chronic” setting, in culture.

In the acute setting, a brief pre-treatment with TGFβ rendered cardiomyocytes incapable of responding to FGF-2 as shown by their blunted ability to upregulate pS262-Cx43; the acute response has “chronic” consequences as it is accompanied by a blunted DNA synthesis and cell proliferation response (Kardami, 1990); see also **Figure 5**. In a more “chronic” scenario, we tested the consequences of inhibiting constitutive TGFβ signal transduction on endogenous pS262-Cx43 levels. The biological effects of TGFβ are transduced by binding to plasma membrane receptors, TGFβRII and TGFβRI, downstream activation/phosphorylation of the regulatory Smads (Smad2 and Smad3), their interaction with Smad4, nuclear translocation and activation of specific gene expression (Massague and Wotton, 2000; Heldin et al., 2009). Inhibition at the level of TGFβR1 was achieved through the use of SB431542, which inhibits TGFRI (ALK5) by acting as a competitive ATP binding site kinase inhibitor (Inman et al., 2002). In cardiomyocytes, which express ALK5, SB431542 is effective in preventing downstream activation (phosphorylation) of Smad2 as shown by (Waghabi et al., 2002), and confirmed here. To specifically target the activity of TGFβRII, which acts as a co-receptor with TGFβRI, we overexpressed a kinase-deficient TGFβRII which has been shown to be effective in inhibiting endogenous TGFβRII in a DN fashion (Sheikh et al., 2004). Finally, Smad2 activation (phosphorylation) was prevented by overexpressing Smad2-DN. All of these inhibitors increased endogenous levels of pS262-Cx43, without affecting total Cx43 protein levels, indicating that early TGFβ signal transduction (engaging the activity of TGFβRII and TGFβRI and resulting in Smad2 phosphorylation) downregulates pS262-Cx43. This may occur by: directly preventing the FGF-2-induced activation of PKCε which acts as an upstream phosphorylating kinase for Cx43; by preventing Cx43 interaction and/or phosphorylation by the PKCε; by activating phosphatase(s) that would cause Cx43 dephosphorylation. There is evidence to support the latter possibility: TGFβ signaling activates protein phosphatase (PP)-2A (Petritsch et al., 2000), which has been reported to target Cx43 for dephosphorylation (Ai et al., 2011). Other potential phosphatases implicated in Cx43 dephosphorylation include PP1 and PP2B (Jeyaraman et al., 2003). There is at present limited information as to potential effects of TGFβ on PKCε activity or expression, or on the PKCε/Cx43 interaction. It is of interest, however, that PKCε is promoting the proliferation of cells, including cardiomyocytes (Kardami et al., 2003), and has an antithetical relationship with TGFβ regarding the control of CD4^+^ T-lymphocyte proliferation (Mirandola et al., 2011).

In parallel to increasing endogenous pS262-Cx43, inhibition of constitutive TGFβ signal transduction increased cardiomyocyte DNA synthesis, providing further validation to the notion that Cx43 phosphorylation at S262 is permissive for mitogenic stimulation. It should, however, be noted that inhibition of constitutive TGFβ signal transduction was unable to reverse the inhibitory effects of overexpressed Cx43. It is possible that overexpressed Cx43 (or Cx43-CT) overwhelm the endogenous cellular machinery (kinases/phosphatases) affected by inhibition of baseline TGFβ signaling. In broad agreement with this notion, we have previously observed that only a small fraction of overexpressed Cx43, or Cx43-CT, become phosphorylated at S262 under normal culture conditions, requiring stimulation with a highly potent PKC activator, phorbol-12-myristate-13-acetate, for Cx43 to become mostly phosphorylated at that site (Dang et al., 2006). A likely explanation therefore for the apparent discrepancy of our findings with endogenous versus overexpressed Cx43 is that any signals (kinases/phosphatases) activated or dis-inhibited by blocking baseline TGFβ signaling may be inadequate to promote or sustain substantial phosphorylation at S262 when Cx43 is present at above-normal levels.

Previous studies using a cardiac cell line, atria-derived HL-1, indicated that Cx43, of unknown phosphorylation status, activated TGFβ transcriptional activity, by activating TGFβRI, and promoting Smad2/3 phosphorylation and nuclear translocation; it was suggested that Cx43-dependent suppression of cardiomyocyte proliferative growth reflected downstream activation of Smad2/3 (Dai et al., 2007). This would position Cx43 expression upstream of TGFβRI-pSmad2 signal transduction, in apparent contrast to findings presented here. We showed that ectopic expression of Cx43 inhibited DNA synthesis regardless of the status of TGFβRI or Smad2/3 activation; in addition neither Cx43 nor Cx43-CT overexpression had any discernible effect on relative pSmad2 levels in our system. Our data therefore suggested that Cx43-mediated inhibition of DNA synthesis is not mediated by downstream activation of TGFβ signals. It is possible that these differences may reflect differences between experimental approaches (gain-of-function in the case of overexpression versus loss-of-function in knock-down studies) and/or cell types, namely the mouse atrial HL-1 cell line versus rat primary ventricular cardiomyocytes used here. Also, as Dai et al. (2007) used TGFβ transcriptional activation as their end-point, there was no information as to how the observed Smad2/3 and/or Cx43 expression changes affected DNA synthesis in their system.

The precise molecular mechanism by which Cx43 and pS262-Cx43 affect proliferative growth remains to be determined. The role of subcellular localization of Cx43 is not clear: Cx43-CT, which, unlike Cx43, localizes to the cytosol and nucleus, retains inhibitory activity (Dang et al., 2003; Kardami et al., 2007), suggesting that localization at cell–cell contact sites and/or gap junction function are not crucial determinants of growth inhibitory activity. Furthermore, phosphorylation at S262 (simulated by expression of S262D-Cx43, or -Cx43-CT) blocked the inhibitory properties of not only Cx43 but also Cx43-CT, without affecting their respective distinct localizations, at plasma membrane versus cytosolic/nuclear sites, in HEK293 cells; unpublished observations and as shown previously (Dang et al., 2003, 2006). It is of interest, however, that Cx43 (Boengler et al., 2005) as well as a C-terminal containing fragment of Cx43 (Kardami et al., 2007) have also been detected in cardiac mitochondria. Mitochondria play an important role in cell cycle regulation (Antico Arciuch et al., 2012), and therefore future studies should examine whether mitochondrial Cx43, and its potential phosphorylation at S262, modulate mitochondrial function in this context.

In conclusion, our studies suggest the following model to describe the relationship between TGFβ and Cx43-mediated growth suppression in cardiomyocytes; **Figure 6**. TGFβ-triggered early signal transduction, involving activation of TGFβRII/RI and phosphorylation of Smad2, reduces relative levels of pS262-Cx43, by either preventing the growth factor-induced PKCε activation,or PKCε/Cx43 interaction, and/or by activating Cx43-targeting phosphatase(s). Cx43 lacking phosphorylation at S262 possesses growth inhibitory activity, blocks growth factor (FGF-2) signals from stimulating cell cycle progression, and mediates or at least contributes to inhibition of DNA synthesis by TGFβ. It has been established that both TGFβ-, and Cx43-, mediated suppression of cell proliferation are achieved by upregulating the cyclin-dependent kinase inhibitors p21/p27 which control cell cycle arrest (Sanchez-Alvarez et al., 2006; Abbas and Dutta, 2009; Bauer et al., 2012), and thus these signals are proposed to be activated downstream of Cx43 lacking phosphorylation at S262. Mitogens, such as FGF-2, overcome/attenuate inhibition of DNA synthesis by Cx43 by promoting activation of PKCε which interacts with, and phosphorylates Cx43 at S262. The ability of FGF-2 to overcome the growth inhibitory effect of Cx43 on cardiomyocytes is likely to depend on expression of appropriate levels of signal-transducing machinery [FGF-2 receptors, active PKCε, inactive phosphatase(s)] to achieve and/or sustain phosphorylation of endogenous Cx43 at S262.

## Conflict of Interest Statement

The authors declare that the research was conducted in the absence of any commercial or financial relationships that could be construed as a potential conflict of interest.
